# Extraction and Identification of Volatile Organic Compounds Emitted by Fragrant Flowers of Three *Tillandsia* Species by HS-SPME/GC-MS

**DOI:** 10.3390/metabo11090594

**Published:** 2021-09-02

**Authors:** Mame-Marietou Lo, Zohra Benfodda, David Bénimélis, Jean-Xavier Fontaine, Roland Molinié, Patrick Meffre

**Affiliations:** 1 UNIV. NIMES, UPR CHROME, CEDEX 1, F-30021 Nîmes, France; mame.lo@unimes.fr (M.-M.L.); zohra.benfodda@unimes.fr (Z.B.); david.benimelis@unimes.fr (D.B.); 2 UMR INRAE 1158 Transfrontaliére BioEcoAgro, BIOlogie des Plantes et Innovation (BIOPI), UPJV, UFR de Pharmacie, 80037 Amiens, France; jean-xavier.fontaine@u-picardie.fr (J.-X.F.); roland.molinie@u-picardie.fr (R.M.)

**Keywords:** *Tillandsia xiphioides*, *Tillandsia crocata*, *Tillandsia caliginosa*, headspace solid phase microextraction (HS-SPME), gas chromatography-mass spectrometry (GC-MS), volatile organic compounds (VOCs), terpenoids, phenylpropanoids, PCA analysis, heatmap

## Abstract

Numerous volatile organic compounds (VOCs) with a large chemical diversity are emitted by plant flowers. They play an important role in the ecology of plants, such as pollination, defense, adaptation to their environment, and communication with other organisms. The *Tillandsia* genus belongs to the Bromeliaceae family, and most of them are epiphytes. The aromatic profile of the *Tillandsia* genus is scarcely described. In this study, we use the headspace solid phase microextraction (HS-SPME) coupled with gas chromatography combined with mass spectrometry (GC-MS) method developed in our laboratory to explore the chemical diversity of the VOCs of fragrant flowers of three species of the genus *Tillandsia*. We were able to identify, for the first time, 66 volatile compounds (monoterpenes, sesquiterpenes, phenylpropanoids, and other compounds). We identified 30 compounds in *T. xiphioides*, 47 compounds in *T. crocata*, and 43 compounds in *T. caliginosa*. Only seven compounds are present in all the species studied. Comparison of the volatile compounds profiles by principal component analysis (PCA) between *T. xiphoides*, *T. crocata*, and *T. caliginosa* species showed a clear difference in the floral emissions of the studied species. Moreover, floral VOCs profiles allowed to differentiate two forms of *T. xiphioides* and of *T. crocata*.

## 1. Introduction

A wide variety of Volatile Organic Compounds (VOCs) are produced and emitted by plants, especially in organs such as flowers, leaves, fruits, and roots. More than 1700 volatile compounds have been identified and can be classified in the following categories: terpenoids, phenylpropanoids, fatty acid and amino acid derivatives, various compounds containing sulfur, as well as furanocoumarins and their derivatives [1,2]. The volatile compounds emitted by flowers generally have a low molecular weight and differ significantly according to the species studied. They play an important role in attracting pollinators, in defense against herbivores, in adaptation to environmental stress, and in communication between flowering plants [3,4]. Floral scent therefore not only has a role in several eco-physiological processes, but also enhances the aesthetic character of ornamental plants and is widely used in the perfume and fragrance industry, in the food industry, and in cosmetics [5]. For all these reasons, the number of scientific studies carried out on floral scent has increased in recent years, motivated by the discovery of new notes useful in perfumery and by the deepening of knowledge in plant physiology [6].

The main analytical method used in the study of VOCs is gas chromatography combined with a mass spectrometer (GC-MS). The analysis of volatile profiles of plants remains a relevant method due to its simplicity and the considerable contribution of data, which allows to differentiate species between them as well as individuals within the same species [5]. Regarding isolation methods for volatile compounds, they can be divided into three categories: steam distillation, solvent extraction, and headspace trapping [7]. Steam distillation and solvent extraction are time-consuming methods requiring the use of large quantities of solvents and can lead to the formation of artifacts either by isolating non-volatile matter from the tissue or by heat-produced re-arrangement [8]. Therefore, soft extraction methods such as headspace trapping are increasingly being used. Headspace solid phase microextraction (HS-SPME) is a widely used method for the extraction of volatile compounds from flowers [9]. It allows the extraction, concentration, and introduction of analytes into the GC inlet in a single step. It is a fast technique, which does not require the use of organic solvents and is characterized by a reduced operating cost due to the reuse of extraction fibers [10].

In our previous study the HS-SPME/GC-MS method was developed to study the VOCs emitted by the flowers of a *Tillandsia* species. Following this, two methods were selected and allowed the identification of 30 volatile compounds from the flowers of *Tillandsia xiphioides* Ker Gawler [11]. For the present study, these two methods were used to identify volatile compounds emitted by the flowers of *Tillandsia crocata* and *Tillandsia caliginosa*. The *Tillandsia* species belong to the Bromeliaceae family. Most of them are epiphytic. The genus *Tillandsia* has a very long history of use as a medicinal plant, especially in traditional medicine [12]. Its main chemical constituents belong to several groups, such as flavonoids, triterpenoids, sterols, and phenylpropanoids, which are known to have various important biological activities [12,13]. However, the volatile composition of *Tillandsia* has been very little studied, although some species have very fragrant flowers. Among these species, *T. crocata* Baker has a short stem, distichous leaves quickly forming dense clumps [14] with small yellow or orange flowers. It is known for its very intense floral scent that attracts male Euglossine bees. For this reason, a study was carried out in 1991 to determine the composition of the floral scent of *T. crocata*, which led to the identification of seven volatile compounds [15]. Concerning *T. caliginosa*, it is a plant with a single stem, sometimes branched, carrying long, succulent leaves covered with grayish white scales. Its small but very odorous flowers have a dark brown color. To our knowledge, no study has yet been done on the composition of the VOCs of *T. caliginosa*.

The objective of this study is to identify, using the HS-SPME/GC-MS method, the VOCs of fragrant flowers of three species of the genus *Tillandsia* with different colors and flower shapes. The flowers of these species are presented in Table 1, these are flowers of two forms of *T. xiphoides* noted low pubescent *xiphioides* (LP-xiphi) and high pubescent *xiphioides* (HP-xiphi), respectively, due to a low and high presence of trichomes on the leaves of these plants; two forms of *T. crocata* are yellow *crocata* and orange *crocata* according to the color of the flower, and one form of *T. caliginosa* presents flowers of dark brown color. 

A principal component analysis (PCA) was performed to profile the variations of compounds of the different species *T. xiphioides*, *T. crocata*, and *T. caliginosa*. Having shown relatively similar profiles, a second PCA as well as a heatmap were performed to better visualize the differences in profiles of *T. crocata* and *T. caliginosa*. This study is the first to compare floral volatile profiles between fragrant *Tillandsia* species and provides additional information on the poorly described floral metabolic network of the genus *Tillandsia.*

## 2. Results

### 2.1. Identification

**Figure 1:**
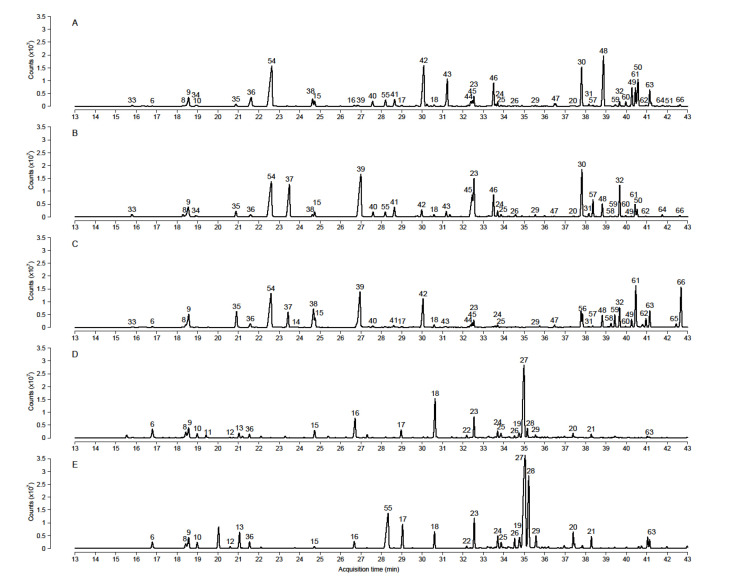
Chromatographic separation was carried out on a DB-5MS column, and the extraction were performed with CAR/PDMS fiber for 65 min at an extraction temperature of 75 °C. (**A**) TIC of orange *crocata*. (**B**) TIC of yellow *crocata*. (**C**) TIC of *T. caliginosa*. (**D**) TIC of LP-xiphi. (**E**) TIC of HP-xiphi. For chromatograms obtained using the first extraction method (low values of temperature and extraction time), see Figure S1 in the Supplementary Material. The identification numbers correspond to those shown in Table 2.

CAR/PDMS fiber with optimal extraction conditions was used to study the volatile compounds emitted from the flowers of three *Tillandsia* species. Through the use of two HS-SPME/GC-MS methods, a total of 66 compounds were identified from the floral emissions of LP-xiphi, HP-xiphi, yellow *crocata*, orange *crocata*, and *T. caliginosa* (Table 2, Figure 1). Among these compounds, only seven—β-myrcene (6), limonene (8), eucalyptol (9), α-terpineol (15), geranyl acetate (18), β-farnesene (23), and α-farnesene (24)—are present in all the species studied. The compounds were categorized into four different classes: monoterpenes, sesquiterpenes, phenylpropanoids, and other compounds including nitrogenous compounds, esters, and fatty acid derivatives (‘others’ family). Qualitative differences in the profile of flower volatiles of these species were observed.

For *T. xiphioides*, a total of 30 volatile compounds were identified in the floral emissions of both forms of this species. The volatile profile is largely dominated by terpenoids, which are 28 of the 30 compounds identified. Indeed, the first extraction method allowed the isolation of more monoterpenes, of which the major ones are limonene (8) and eucalyptol (9) for LP-xiphi and β-linalool (13) for HP-xiphi. With the use of higher values of temperature and extraction time, the second extraction method allowed the isolation of higher molecular weight compounds. The major ones are geranyl acetate (18) and nerolidol (27) for LP-xiphi and indole (55), nerolidol (27), and denderalisin (28) for HP-xiphi. Among the compounds identified in both forms of *T. xiphioides*, only indole (55) and methyl palmitate (63) are present in the HP-xiphi form and not in the LP-xiphi form.

For *T. crocata*, 47 volatile compounds were identified in the floral emissions of both forms of this species. This completes the list of seven VOCs, consisting of benzaldehyde (33) (3%), benzyl acetate (37) (9%), eucalyptol (9) (16%), limonene (8) (3%), methyl salicylate (38) (2%), *E*-β-ocimene (10) (48%), and phenylethyl acetate (39) (8%), identified by Gerlach and Schill in 1991 [15]. In contrast to *T. xiphioides*, where no compounds of the phenylpropanoid family were identified, for *T. crocata*, these compounds are very present. Indeed, phenylpropanoids are secondary metabolites derived from phenylalanine by the shikimate biosynthetic pathway. Only compounds forming an aldehyde, an alcohol, or an alkane/alkene by reduction at C9 position, or ethers and esters by addition of an alkyl group on hydroxyl groups or carboxyl group, can be volatile [16]. Thus, among the identified compounds, the first extraction method allowed us to isolate mainly benzyl acetate (37), phenylethyl acetate (39), eucalyptol (9), and limonene (8) for the yellow *crocata* and limonene (8) and eucalyptol (9) for the orange *crocata*. The second extraction method allowed us to isolate more compounds and the major ones are farnesol (30), phenylethyl acetate (39), isoeugenol (45), methyl nicotinate (54), benzyl acetate (37), β-farnesene (23), and farnesyl acetate (32) for yellow *crocata* and benzyl benzoate (48), methyl nicotinate (54), eugenol (42), farnesol (30), benzyl salicylate (50), and methyl eugenol (43) for orange *crocata*. Moreover, for the species *T. crocata*, some VOCs are present only in one of the two forms of *crocata*, such as α-bergamotene (22) and benzyl acetate (37), which are present only in yellow *crocata*, as well as α-thujene (1), β-pinene (5), geraniol (16), cinnamyl acetate (44), phenethyl salicylate (51), and methyl palmitate (63) have been identified only in orange *crocata.*

For *T. caliginosa*, 43 volatile compounds were identified for the first time in floral emissions. The volatile profile is quite similar to that of the species *T. crocata*; however, there are fewer sesquiterpenes, which are only five in *T. caliginosa*, and the compounds belonging to the ‘others’ family are more numerous in *T. caliginosa* than in *T. crocata* and in *T. xiphioides*. The first extraction method allowed us to isolate mostly limonene (8), eucalyptol (9), and phenylethyl acetate (39), while the second method allowed us to extract mostly hexadecyl ethanoate (66), 1-hexadecanol (61), phenylethyl acetate (39), and methyl nicotinate (54) from the flowers of *T. caliginosa.*

### 2.2. Chemometrics Analysis of Volatile Organic Compounds

**Figure 2:**
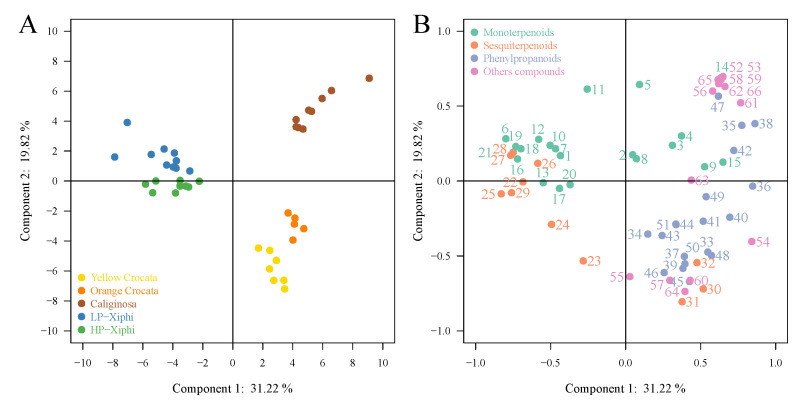
PCA based on VOCs emitted from yellow crocata, orange crocata, caliginosa, HP-xiphi and LP-xiphi: (**A**) Score plot of PC1 versus PC2 scores. (**B**) Loading plot of PC1 and PC2 contributing volatile compounds.

In order to identify volatile compounds that contribute to differences or similarities between the species studied, data on the 66 identified volatiles were analyzed using principal component analyses (PCA). PCA is a multivariate analysis that allows the extraction of important information from a data table in which observations are described by several intercorrelated dependent variables and represents them as a set of new orthogonal variables called principal components. In Figure 2A, it is shown that the first principal component (PC1) contributes to 32.7%, and the second principal component (PC2) contributes to 17.29% of the total variance in the floral volatile peak area data. On this same figure, we can see three distinct groups; indeed, the flowers of the two forms of *T. xiphioides* are clustered together, the flowers of the two forms of *T. crocata* are clustered together, and the flowers of *T. caliginosa* form the third group. This shows a high degree of clustering of flowers belonging to the same species, thus suggesting a unique floral volatile profile for each species of the genus *Tillandsia*. PC1 allowed discrimination of LP-xiphi and HP-xiphi flowers that are clearly separated from yellow *crocata*, orange *crocata*, and *caliginosa* flowers. This separation is due to higher intensities of monoterpenoids and sesquiterpenoids in the floral emissions of both forms of *T. xiphioides* (Figure 2B). PC2 showed a difference in floral volatiles of *T. caliginosa* compared to the two forms of *T. crocata*. This was due to high phenylpropanoids intensities in both forms of *T. crocata* while *T. caliginosa* showed higher intensities of other compounds.

**Figure 3:**
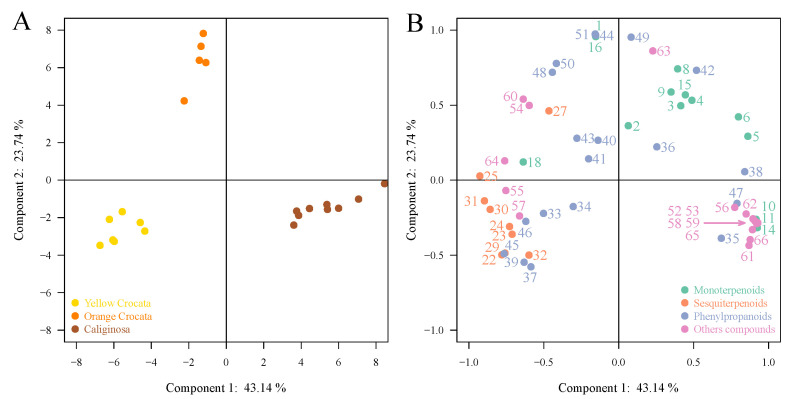
Chemometric analysis of 57 VOCs emitted by the flowers of yellow crocata, orange crocata and T. caliginosa. (**A**) Score plot of PC1 versus PC2 scores. (**B**) Loading plot of PC1 and PC2 contributing volatile compounds.

**Figure 4:**
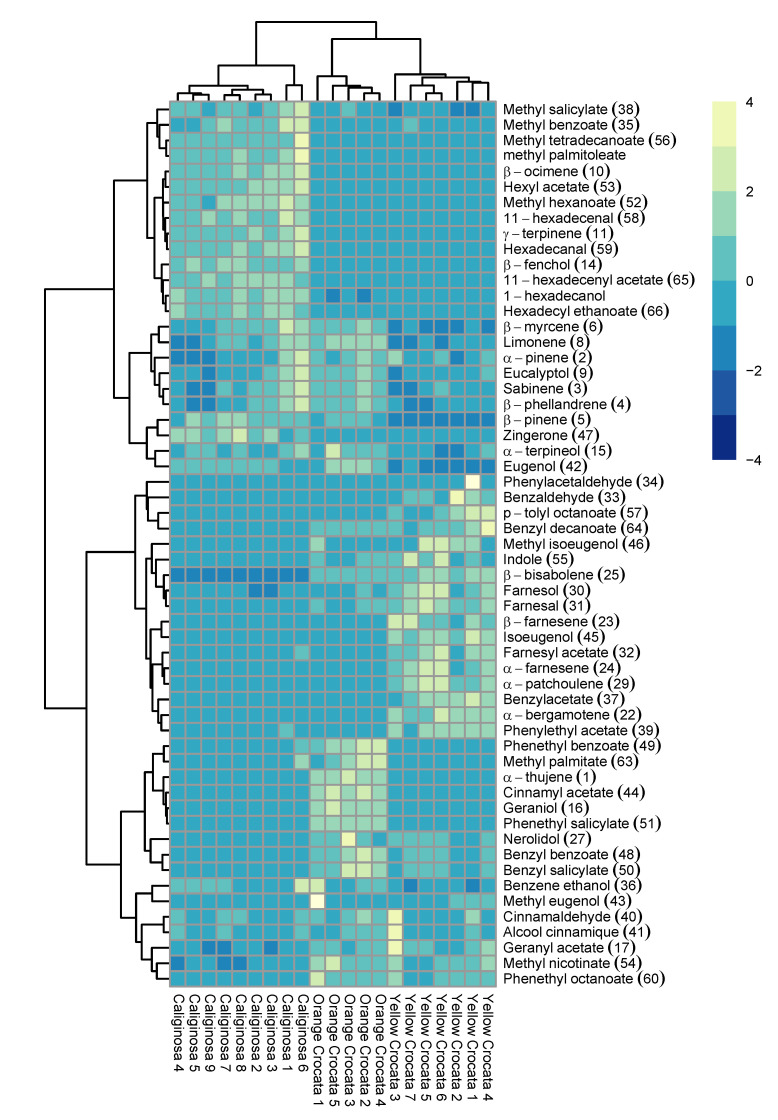
Heatmap, the levels of each VOC in the three types of flowers were normalized in the range of −4 to 4. Blue (−4) and yellow (4) represent the lowest and highest levels, respectively.

To better visualize the differences in volatile profiles between flowers of the two forms of *T. crocata* and those of *T. caliginosa*, a second PCA as well as a heatmap were performed (Figure 3 and Figure 4). The first two components of the PCA explained 43.14% and 23.74% of the variation, explaining approximately 66% of the combined variance (Figure 3A). We can see that the two forms of *T. crocata* have relatively different floral volatile profiles due to their higher intensities of sesquiterpenoids and phenylpropanoids, such as β-farnesene (23), farnesyl acetate (32), benzylacetate (37), and phenylethyl acetate (39), which have higher intensities in floral emanations of yellow *crocata* compared to orange *crocata* (Figure 3B). While compounds such as benzyl benzoate (48), eugenol (42), methyl eugenol (43), benzyl salicylate (50), and phenethyl benzoate (49) are more abundant in orange *crocata* flowers, *T. caliginosa* flowers are grouped on the right due to higher intensities of other compounds, such as 1-hexadecanol (61), hexadecyl ethanoate (66), methyl tetradecanoate (56), hexadecanal (59), and some phenylpropanoids, such as methyl benzoate (35) and methyl salicylate (38).

This is confirmed by the heatmap (Figure 4); indeed, it is shown that the 57 volatile compounds detected in the floral emissions of *T. caliginosa* and in the two forms of *T. crocata*, are grouped in four large groups in the dendogram of the heatmap. The 14 compounds of group 1, consisting of methyl salicylate, methyl benzoate, methyl tetradecanoate, methyl palmitoleate, β-ocimene, hexyl acetate, methyl hexanoate, 11-hexadecenal, γ-terpinene, hexadecanal, β-fenchol, 11-hexadecenyl acetate, 1-hexadecanol, and hexadecyl ethanoate, are more abundant in the flowers of *T. caliginosa*. The 10 volatile molecules of Group 2, consisting of β-myrcene, limonene, α-pinene, eucalyptol, sabinene, β-phellandrene, β-pinene, zingerone, α-terpineol, and eugenol, are relatively abundant in the floral emissions of *T. caliginosa* and highly present in orange *crocata* flowers compared to yellow *crocata*. Group 3, consisting of 17 compounds, is formed mostly by sesquiterpenes and phenylpropanoids, which are abundant in yellow *crocata* flowers, while Group 4 is formed by 16 volatile compounds, which are abundant in orange *crocata* floral emissions.

## 3. Discussion

The identification of VOCs of three species of the genus *Tillandsia* was achieved through the use of the HS-SPME/GC-MS method. By using the CAR/PDMS fiber and two extraction methods with different values of extraction time and temperature developed in a previous study, the majority of the volatile compounds emitted by the flowers of *Tillandsia* species were extracted efficiently. Indeed, it was shown that the CAR/PDMS fiber allowed to extract the maximum of volatile compounds from *T. xiphioides* flowers compared to PDMS/DVB and DVB/CAR/PDMS fibers; moreover, considering the volatility of analytes affecting the optimal conditions of temperature and duration of extraction, it is necessary to use two extraction methods in order to trap the majority of volatiles emitted by *Tillandsia* flowers [11]. After the GC-MS analysis, the profiles of the volatiles emitted by the flowers of the different species studied were compared qualitatively. This is the first time that a comparison of the VOCs emitted by different fragrant flowers of the genus *Tillandsia* has been performed. The results showed a difference in volatile profiles between the flowers of the species *T. xiphioides*, *T. crocata*, and *T. caliginosa*. Indeed, the flowers of the two forms of *T. xiphioides* present a profile of volatile compounds much more enriched in monoterpenes and sesquiterpenes, compared to the flowers of *T. crocata* and *T. caliginosa*, which present relatively more similar profiles. This could be confirmed by a morphological difference but also by a difference in pollinator type between *T. xiphioides* species and those of *T. crocata* and *T. caliginosa*. Indeed, it is shown that *T. caliginosa* and *T. crocata* are morphologically closer—these species present the same types of foliage and flowers; moreover the old name given to *T. caliginosa* is *T. crocata var. tristis* [17]. This difference in the composition of floral volatiles could also be confirmed by the attraction of different pollinators [18] for *T. crocata*, whose floral odor attracts Euglossine bees [15] and *T. xiphioides*, which is considered sphingophilous [19]. Indeed, it is shown that plants pollinated by the same ecological guild show similar visual and olfactory signals [20].

Even though it is not the only characteristic that can play a role in the attraction of pollinators, the floral odor constituted by a complex mixture of volatile compounds belonging to different chemical families plays a very important role in the stimulation and attraction of pollinators. These floral volatile compounds can be classified in the family of terpenoids, phenylpropanoids/benzenoids, and fatty acid derivatives, according to their biosynthetic pathways. Found predominantly in the flowers of both forms of *T. xiphioides*, terpenoids are derived from two precursors with five carbons isopentenyl diphosphate (IPP) and its allylic isomer dimethylallyl diphosphate (DMAPP) and represent the largest class of floral volatile compounds. The latter are synthesized in plants from two independent pathways: the mevalonic acid pathway, where synthesis is carried out in the cytosol, giving rise to precursors of volatile sesquiterpenes (C_15_); and the methyl-erythritol phosphate pathway, where synthesis is carried out in the plastids mainly responsible of the formation of monoterpenes (C_10_) and volatile diterpenes (C_20_) [1]. Regarding the odor of this group of compounds, monoterpenoid hydrocarbons generally have a spicy and/or resinous odor, oxygenated monoterpenes have a sweet or citrus odor, and sesquiterpenes have a green and floral odor; however, diterpenes are rarely present in floral fragrances due to their low volatility [7]. Although close in the composition of floral volatiles, the two forms of *T. xiphioides* present, nevertheless, a slight difference in the intensities of certain compounds, which can affect the floral odor and lead to a difference in odor for the two forms of this species [11]. 

Found mainly in the flowers of both forms of *T. crocata*, phenylpropanoids represent the second class of VOCs emitted by plants. The first molecule of the phenylpropanoid pathway is phenylalanine, which is derived from the shikimate biosynthetic pathway. The deamination of this molecule by phenylalanine ammonia-lyase (PAL) forms the phenylpropanoid skeleton producing trans-cinnamic acid. All phenylpropanoid compounds are derived from trans-cinnamic acid by a succession of hydroxylation, methylation, and reduction steps [21]. Among the phenylpropanoids identified in this study, benzylacetate and phenylethyl acetate were found mainly in the floral emissions of yellow *crocata*. Benzyl acetate is a compound with a pleasant odor and is commonly found in plants such as jasmine, hyacinth, gardenia, and azalea, and is thought to be formed from the oxidation of cinnamoyl CoA [7,8,22]. Phenylethyl acetate is widely used in the cosmetic, food, and pharmaceutical industries for its pleasant rose smell. It is present in rose essential oils and is the main aromatic volatile ester emitted by rose flowers. Traditionally, this compound is obtained by acetylation of phenylethyl alcohol; moreover, the acetylation of aromatic alcohols catalyzed by alcohol acetyltransferase leads to volatile acetate esters in plants [23,24]. Other phenylpropanoids, such as eugenol, methyl eugenol, benzyl benzoate, phenethyl benzoate, and benzyl salicylate, were very present in the floral emissions of orange *crocata*. In the phenylpropanoid biosynthetic pathway, eugenol and isoeugenol are obtained from coniferyl acetate through eugenol synthase and isoeugenol synthase. Subsequently, a methylation reaction of eugenol, catalyzed by an *O*-methyltransferase, allows us to obtain methyl eugenol [21]. Eugenol and methyl eugenol are major constituents of several essential oils, such as clove, basil, cinnamon, lavender, and jasmine essential oils. Eugenol is widely used in perfumery due to its powerful spicy odor, and methyl eugenol is widely used as a flavoring agent in food products [25,26,27]. In plants such as *Clarkia breweri* and *Petunia hybrida*, benzyl benzoate and phenethyl benzoate are obtained from benzyl alcohol and phenylethanol, respectively, through benzoyl-CoA: benzyl alcohol/phenylethanol benzoyltransferase [28,29]. In addition to its activity in the treatment of scabies and lice, benzyl benzoate is used as a fixative in perfumery [26]. Benzyl salicylate is the benzyl ester of salicylic acid, it is widely used in the cosmetics industry and is used in the composition of many perfumes [30]. Volatile aromatic esters generally contribute to a sweet fruity smell [31]. These results show that there is a slight difference in the floral volatile profiles of the two forms of *T. crocata*, especially in the intensities of some volatile compounds. This difference could be associated with the difference in floral colors of the two forms of this species. Indeed, it was shown that plants of the same species having a variation of floral color also presented a variation in the composition of floral volatiles. For example, a study on orchids showed that the color morphs of *O. simia* presented different volatile profiles; the white flowers emitted more benzenoid compounds and lipid products than the purple flowers [32]. However, there are studies that did not find a clear difference in the odor chemistry of color morphs of the same species [33,34]. Thus, even if the slight difference in volatile profiles of yellow and orange *crocata* flowers could be associated with the difference in flower colors, it could also be due to natural selection by pollinators or mutations in genetic drift or metabolic pathways. 

In *T. caliginosa*, floral emissions showed, in addition to phenylpropanoids, higher intensities of compounds of the ‘others’ family than in the ‘others’ species. Indeed, compounds such as 1-hexadecanol, hexadecyl ethanoate, methyl tetradecanoate, and hexadecanal are very present in *T. caliginosa*. These compounds are derivatives of fatty acids, which constitute another large family of volatile compounds. They are produced following a series of reactions catalyzed by lipoxygenases, hydroperoxide lyases, isomerases, and dehydrogenases from polyunsaturated fatty acids and phospholipids [1]. They generally bring a waxy and oily note to the floral scent of the plants. It should also be noted that the flowers of both forms of *T. crocata* as well as *T. caliginosa* have shown a high intensity of methyl nicotinate. The latter is a vasodilator, which is used in topical preparations as a rubefacient in case of muscle pain [35].

## 4. Materials and Methods

### 4.1. Plant Material and Chemicals

All the flowers of the *Tillandsia* species used for this study come from the *Tillandsia* PROD plant nursery located in Le Cailar (Occitanie, France) and were harvested from February to May 2020. We used two forms of *T. xiphioides*, named LP-xiphi and HP-xiphi due to low or high presence of trichomes on the leaves of the plants; two forms of *T. crocata*, named yellow *crocata* and orange *crocata* with yellow and orange colored flowers, respectively; and one form of *T. caliginosa* species. For *T. xiphioides*, 10 plants of each form were used, providing 2–3 flowers each. For *T. crocata*, 7 plants (one flower per plant) of the yellow *crocata* form and 5 plants of the orange *crocata* form providing 2 flowers each were used. For *T. caliginosa*, 8 plants were used and 5 of them provided 2 flowers.

The chemicals used for the identification of the compounds were ordered from Sigma-Aldrich (St. Louis, MO, USA): α-pinene (98.5% purity), sabinene (75%), β-pinene (98.5%), β-myrcene (90%), limonene (99%), eucalyptol (99%), β-ocimene (95.4%), γ-terpinene (98.5%), β-fenchol (99%), β-linalool (97%), α-terpineol (90%), geraniol (99%), geranyl acetate (99%), nerolidol (98.5%), farnesol (95%), benzaldehyde (99.5%), phenylacetaldehyde (95%), methyl benzoate (99.5%), benzylacetate (99.7%), methyl salicylate (99%), phenylethyl acetate (97%), alcool cinnamique (97%), eugenol (99.6%), methyl eugenol (98%), isoeugenol (99%), benzyl benzoate (98%), hexyl acetate (99.7%), and methyl nicotinate (98%).

### 4.2. HS-SPME Conditions

In our previous study, the Carboxen/Polydimethylsiloxane (CAR/PDMS) 75 µm fiber was determined to be the most suitable for extracting the maximum amount of volatile compounds emitted by *T. xiphioides* flowers, so this fiber was used for the present study. The CAR/PDMS and the SPME device were ordered from Supelco (Bellefonte, PA, USA). Prior to use, the fiber was conditioned according to the manufacturer’s recommendations.

Extractions were performed by placing a flower in a 20 mL amber vial sealed with a polytetrafluoroethylene (PTFE) septum-lined cap. For each *Tillandsia* flower, two successive extractions were performed: a first extraction method of 20 min at a temperature of 30 °C and a second extraction method of 65 min at 75 °C. These extraction methods provide a global view of the volatile compounds present in *Tillandsia* flowers and were retained after the optimization of the SPME method performed during our previous study. For both extraction methods, the equilibrium and desorption times are, respectively, 7.5 and 4 min. 

### 4.3. Instrumentation and GC-MS Conditions

The analysis was performed on an Agilent 7890 B gas chromatograph coupled with an Agilent 5977 A mass spectrometer (Agilent Technologies, Santa Clara, CA, USA) with an MPS autosampler, a thermal desorption unit (TDU), and a cooled injection system (CIS) (Gerstel, Mülheim, Germany). MSD Chemstation version F.01.00 data acquisition software (Agilent Technologies, Santa Clara, CA, USA) was used to program the GC-MS. After extraction, desorption was performed thermally in the TDU at the recommended temperature of 300 °C for CAR/PDMS. After desorption, the injection was performed in split mode; the analytes were focused on the CIS at −10 °C for 2 min, then brought to 250 °C at a heating rate of 12 °C per second and maintained for 2.5 min. A DB-5MS capillary column (5% diphenyl cross-linked 95% dimethylpolysiloxane, 30 m × 0.25 mm × 0.25 μm) (Agilent Technologies, Santa Clara, CA, USA) was used and the separation conditions were as follows: initial column temperature of 40 °C for 2 min, then increased by 4 °C/min to 130 °C for 1 min, then increased by 7 °C/min to 230 °C, where it was maintained for 4 min. High purity Helium (99.999%) was used as the carrier gas at a flow rate of 1.2 mL per minute. The temperature of the transfer line was set at 250 °C, and the temperature of the ion source at 230 °C. The ions were generated by a 70 eV electron beam. The mass range was scanned from *m*/*z* 33 to 500 Da.

### 4.4. Identification

MassHunter Qualitative Analysis version B.06.00 (Agilent Technologies, Santa Clara, CA, USA) was used to integrate the majority peaks of the chromatograms, representing 95% of the peaks of the Total Ion Chromatogram (TIC) and being above the analytical noise. First, the volatile compounds were identified by comparing their mass spectra to those of compounds in the commercial databases, National Institut of Standard and Technologies (NIST) and Wiley7 (R > 800). Then, the retention indices of the compounds were calculated relative to the n-alkanes (C8–C20) and compared to those of compounds in the NIST online database (https://webbook.nist.gov/chemistry/cas-ser.html, accessed on 27 August 2021). Finally, the identifications were confirmed by injecting the available standards (mentioned above) into the GC-MS based on the comparison of mass spectra and retention times. For the standard solution, 0.2 µL of each standard was added in 2 mL of hexane, and the solvent was subsequently evaporated with nitrogen. The analysis was performed under the same conditions as for the flowers.

### 4.5. Statistical Analysis

For statistical analysis, peak area values of the total ion chromatograms were measured with MassHunter and transferred to Excel (Microsoft Excel 2013, version 15.0.5363.1000). Principal component analysis (PCA) was performed using SIMCA-P software (version 15.15, Umetrics, Umea, Sweden) and a heatmap with the R software (version 4.0.3, company Foundation for Statistical Computing, Vienna, Austria) based on an univariate scaling method. For this purpose, for each plant providing two flowers (the plants of orange *crocata* and the plants of *T. caliginosa*), an average of the areas of the peaks of the compounds was calculated in Excel. Odor characteristics were obtained from the “The Good Scents” company network database (www.thegoodscentscompany.com, accessed on 27 August 2021). Heatmap data was clustered (Ward’s method was used to form hierarchical clustering) and visualized (using the pheatmap-package, version 1.0.12).

## 5. Conclusions

This study allowed the identification of 66 volatile compounds in three *Tillandsia* species with different colors and flower shapes. This identification was performed using the HS-SPME/GC-MS technique, where the use of two extraction methods, developed in a previous study, were necessary in order to efficiently extract the majority of floral volatiles. A comparison of the volatile compound profiles by PCA between *T. xiphioides*, *T. crocata*, and *T. caliginosa* species showed a difference in the floral emissions of the studied species. Indeed, the floral emissions of *T. xiphioides* were richer in terpenoids, those of T. crocata were enriched in phenylpropanoids, and those of *T. caliginosa* presented more compounds of the ‘others’ family, including fatty acid derivatives and nitrogenous compounds. In addition, a slight intraspecific difference was also observed in *T. crocata*, particularly variations in the intensities of some VOCs of yellow and orange *crocata*. These variations in floral volatile profiles between different *Tillandsia* species and between different forms of the same species could be associated with several phenomena, such as natural selection by pollinators, morphological and/or color differences, mutations in metabolic pathways, or genetic drift. The differences and variations observed in the VOCs profiles can explain the differences in the floral odor of these species and forms. This study provides additional information on the composition of floral VOCs of *Tillandsia* species and helps to better understand the diversity within the genus *Tillandsia*.

## Figures and Tables

**Table 1:** Characteristics of the flowers of three species of the genus *Tillandsia* used in this study.

Name	LP-Xiphi	HP-Xiphi	Yellow *crocata*	Orange *crocata*	*T. caliginosa*
	** 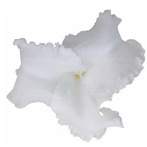 **	** 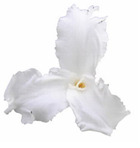 **	** 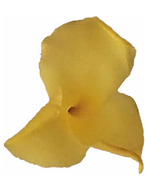 **	** 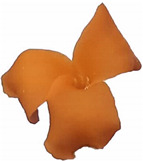 **	** 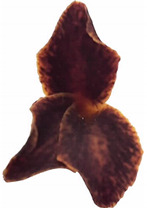 **
Height * (mm)	50	50	20	15	15
Breadth * (mm)	20	20	5	5	5
Odor level	Scented	Scented	Scented	Scented	Scented

* = average values.

**Table 2:** Identification of volatile compounds from floral emissions of *T. xiphioides*, *T. crocata*, and *T. caliginosa*.

#	Family	Compound	RT (min)	Composition (%)				
				Yellow *crocata*	Orange *crocata*	*T. Caliginosa*	LP-Xiphi	HP-Xiphi
1	M	α-thujene ^b, d^	14.15	nd	0.2	nd	0.3	nd
2	M	α-pinene ^c, d^	14.46	0.3	0.3	0.3	0.5	nd
3	M	Sabinene ^c, d^	16.05	0.2	0.3	0.4	0.3	nd
4	M	β-phellandrene ^b, d^	16.23	0.3	0.5	0.6	0.5	nd
5	M	β-pinene ^c, d^	16.46	nd	0.2	0.4	0.3	nd
**6**	**M**	**β-myrcene ^c, d^**	**16.78**	**0.5**	**1.2**	**1.6**	**4.7**	** 6.6 **
7	M	α-phellandrene ^b, d^	17.51	nd	nd	nd	0.7	nd
**8**	**M**	**Limonene ^c, d^**	**18.52**	**6.4**	**11.4**	**10.8**	**10.1**	** 11.5 **
**9**	**M**	**Eucalyptol ^c, d^**	**18.66**	**8.1**	**11.5**	**12.1**	**7.2**	** 7.9 **
10	M	β-ocimene ^c, d^	18.98	nd	nd	0.4	18.8	9.3
11	M	γ-terpinene ^c, d^	19.5	nd	nd	0.2	0.3	nd
12	M	Terpinolene ^b, d^	20.57	nd	nd	nd	0.3	nd
13	M	β-linalool ^c, d^	21.02	nd	nd	nd	1.6	16.1
14	M	β-fenchol ^c, d^	23.8	nd	nd	0.3	nd	nd
**15**	**M**	**α-terpineol ^c, d^**	**24.71**	**1.2**	**1.9**	**2.1**	**0.8**	** 0.9 **
16	M	Geraniol ^c, d^	26.64	nd	0.3	nd	1.2	1.1
17	M	Methyl geranate ^b, e^	29.02	nd	nd	nd	nd	1.9
**18**	**M**	**Geranyl acetate ^c, e^**	**30.59**	**0.3**	**0.3**	**0.2**	**19.6**	**9.4**
19	M	Geranyl butyrate ^b, e^	34.75	nd	nd	nd	1.5	1.3
20	M	Geranyl tiglate ^b, e^	37.39	nd	nd	nd	nd	1.4
21	M	Geranyl hexanoate ^b, e^	38.27	nd	nd	nd	0.9	1.2
22	S	α-bergamotene ^b, e^	32.16	0.2	nd	nd	0.5	0.8
**23**	**S**	**β-farnesene ^b, e^**	**32.55**	**5.5**	**1.2**	**1.0**	**3.3**	** 3.5 **
**24**	**S**	**α-farnesene ^b, e^**	**33.69**	**1.2**	**0.4**	**0.4**	**1.2**	** 1.2 **
25	S	β-bisabolene ^b, e^	33.86	0.3	0.2	nd	0.8	1.7
26	S	α-bisabolene ^b, e^	34.52	nd	nd	nd	0.5	0.9
27	S	Nerolidol ^c, e^	34.98	0.3	0.4	nd	18.7	14.6
28	S	Denderalasin ^b, e^	35.17	nd	nd	nd	4.7	5.0
29	S	α-patchoulene ^b, e^	35.55	0.4	nd	nd	0.7	0.8
30	S	Farnesol ^c, e^	37.81	9.3	4.3	1.8	nd	nd
31	S	Farnesal ^b, e^	38.16	0.6	0.3	nd	nd	nd
32	S	Farnesyl acetate ^b, e^	39.67	4.5	0.5	1.3	nd	nd
33	P	Benzaldehyde ^c, d^	15.8	0.5	0.2	0.2	nd	nd
34	P	Phenylacetaldehyde ^c, d^	18.92	2.3	0.3	nd	nd	nd
35	P	Methyl benzoate ^c, d^	20.89	0.8	0.3	2.2	nd	nd
36	P	Benzene ethanol ^b, d^	21.57	0.9	1.3	1.5	nd	nd
37	P	Benzylacetate ^c, d^	23.43	5.5	nd	1.3	nd	nd
38	P	Methyl salicylate ^c, d^	24.63	0.6	1.1	2.4	nd	nd
39	P	Phenylethyl acetate ^c, d^	26.87	14.2	nd	2.5	nd	nd
40	P	Cinnamaldehyde ^b, e^	27.5	0.5	0.6	0.5	nd	nd
41	P	Alcool cinnamique ^c, e^	28.61	1.2	1.2	0.8	nd	nd
42	P	Eugenol ^c, e^	29.96	0.9	14.5	12.3	nd	nd
43	P	Methyl eugenol ^c, e^	31.18	0.4	1.0	nd	nd	nd
44	P	Cinnamyl acetate ^b, e^	32.37	nd	0.7	nd	nd	nd
45	P	Isoeugenol ^c, e^	32.45	5.1	0.6	0.9	nd	nd
46	P	Methyl isoeugenol ^b, e^	33.49	2.8	0.8	nd	nd	nd
47	P	Zingerone ^b, e^	36.5	nd	0.5	2.7	nd	nd
48	P	Benzyl benzoate ^c, e^	38.83	4.5	8.9	1.8	nd	nd
49	P	Phenethyl benzoate ^b, e^	40.24	0.2	5.1	1.4	nd	nd
50	P	Benzyl salicylate ^b, e^	40.51	1.7	5.1	nd	nd	nd
51	P	Phenethyl salicylate ^a, e^	42.09	nd	0.2	nd	nd	nd
52	O	Methyl hexanoate ^b, d^	14.24	nd	nd	0.3	nd	nd
53	O	Hexyl acetate ^c, d^	17.58	nd	nd	0.2	nd	nd
54	O	Methyl nicotinate ^c, d^	22.59	13.3	15.2	10.8	nd	nd
55	O	Indole ^b, e^	28.22	1.2	0.6	nd	nd	1.1
56	O	Methyl tetradecanoate ^b, e^	37.85	nd	nd	1.7	nd	nd
57	O	p-tolyl octanoate ^b, e^	38.36	1.1	0.2	nd	nd	nd
58	O	11-hexadecenal ^b, e^	39.24	nd	nd	0.5	nd	nd
59	O	Hexadecanal ^b, e^	39.43	nd	nd	1.3	nd	nd
60	O	Phenethyl octanoate ^b, e^	39.97	0.3	0.4	nd	nd	nd
61	O	1-hexadecanol ^b, e^	40.42	2.0	1.0	10.2	nd	nd
62	O	Methyl palmitoleate ^a, e^	40.96	nd	nd	1.2	nd	nd
63	O	Methyl palmitate ^a, e^	41.14	nd	4.3	1.7	nd	1.7
64	O	Benzyl decanoate ^a, e^	41.76	0.3	0.2	nd	nd	nd
65	O	11-hexadecenyl acetate ^a, e^	42.43	nd	nd	0.7	nd	nd
66	O	Hexadecyl ethanoate ^a, e^	42.66	0.5	nd	6.9	nd	nd

# = compound number, in bold: compounds present in all species, M = Monoterpene, S = Sesquiterpene, P = Phenylpropanoid, O = Other, nd = not detected, a = identification performed by comparing the mass spectrum with that of the NIST library, b = identification performed by comparing the mass spectrum with that of the NIST library and by comparison of RI (retention index) with RI of published literatures and online library (https://webbook.nist.gov/chemistry/cas-ser.html, accessed on 28 August 2021), c = identification performed by comparing the mass spectrum with that of the NIST library, by comparison of RI (retention index) with RI of published literatures and online library and by comparison of retention time and mass spectrum of the authentic standard, d = efficient extraction with the first method, low values of temperature and extraction time (30 °C and 20 min), e = efficient extraction with the second method, high values of temperature and extraction time (75 °C and 65 min). For the complete table, see Table S1 in the Supplementary Materials.

## Data Availability

The data presented in this study are available within the article and Supplementary Materials. Any other data are available upon request from the corresponding author.

## Supplementary Materials

The following are available online at https://www.mdpi.com/article/10.3390/metabo11090594/s1, Figure S1: Chromatograms obtained using the first extraction method (low values of temperature and extraction time), Table S1: Complete table of the identification of volatile compounds from floral emissions of *T. xiphioides*, *T. crocata* and *T. caliginosa*.
