# Supplementary material for: Extraction and Identification of Volatile Organic Compounds Emitted by Fragrant Flowers of Three Tillandsia Species by HS-SPME/GC-MS
Source: Metabolites. 2021 Sep 2;11(9):594. doi: 10.3390/metabo11090594 (PMC8471741; doi:10.3390/metabo11090594)
Supplement: Supplementary file 1 [file metabolites-11-00594-s001.zip › metabolites-1320187-SI.pdf]

metabolites

MDPI

Supplementary Material

Extraction and Identification of Volatile Organic Compounds Emitted by Fragrant Flowers of Three *Tillandsia* Species by HS-SPME/GC-MS

Mame-Marietou Lo [1], Zohra Benfodda [1], David Bénimélis [1], Jean-Xavier Fontaine [2], Roland Molinié [2] and Patrick Meffre [1,*]

[1] UNIV. NIMES, UPR CHROME, CEDEX 1, F-30021 Nîmes, France; mame.lo@unimes.fr (M.-M.L.); zohra.benfodda@unimes.fr (Z.B.); david.benimelis@unimes.fr (D.B.)
[2] UMR INRAE 1158 Transfrontaliére BioEcoAgro, BIOlogie des Plantes et Innovation (BIOPI), UPJV, UFR de Pharmacie, 80037 Amiens, France; jean-xavier.fontaine@u-picardie.fr (J.-X.F.); roland.molinie@u-picardie.fr (R.M.)
* Correspondence: patrick.meffre@unimes.fr

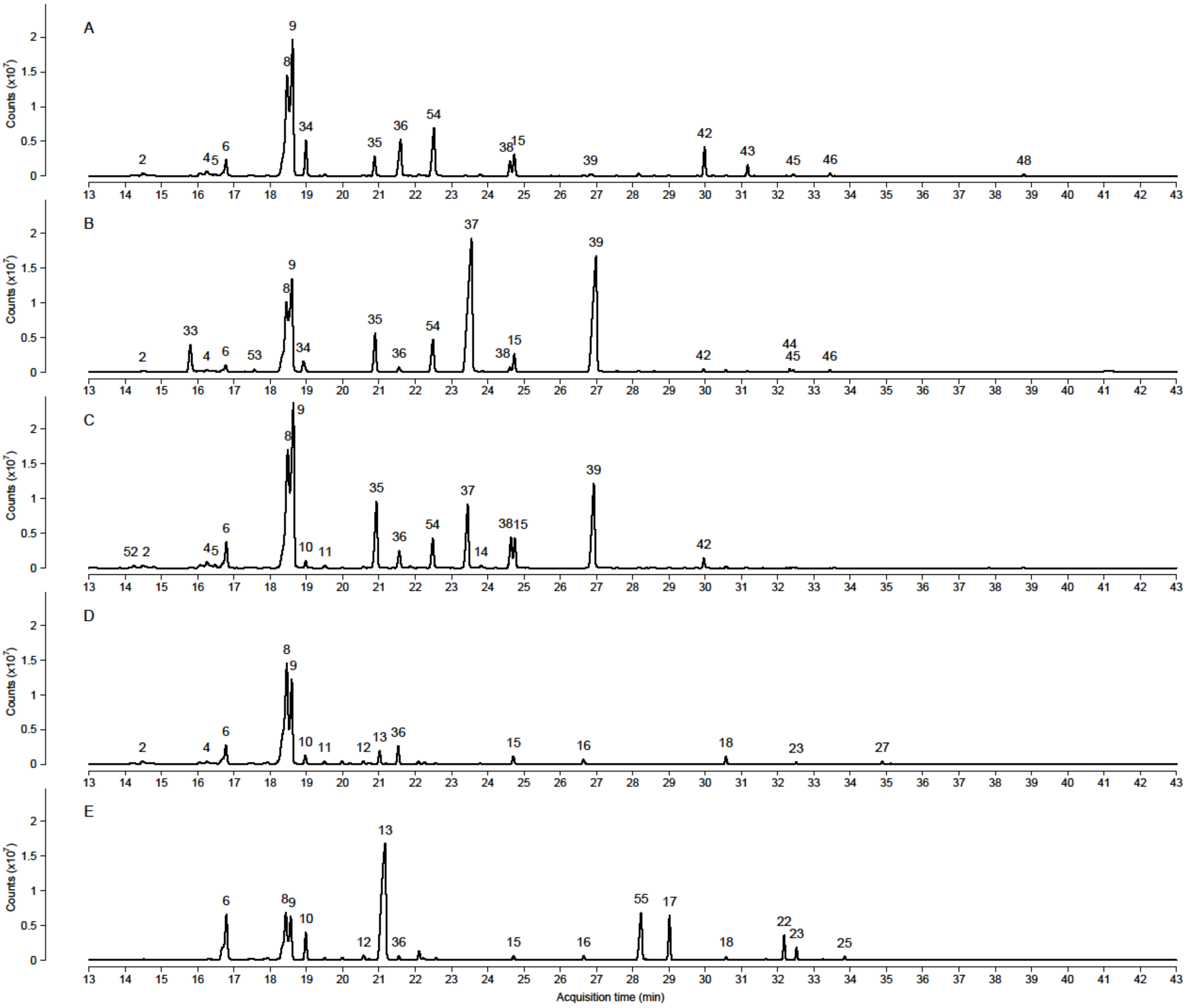

Figure S1: Chromatograms obtained using the first extraction method (low values of temperature and extraction time). Chromatographic separation was carried out on a DB-5MS column and the extraction were performed with CAR/PDMS fiber for 20 minutes at an extraction temperature of 30 °C. (A) TIC of orange *crocata*. (B) TIC of yellow *crocata*. (C) TIC of *T. caliginosa*. (D) TIC of LP-xiphi. (E) TIC of HP-xiphi. The identification numbers correspond to those shown in Table 2 and in Table S1.

Table S1. Complete table of the identification of volatile compounds from floral emissions of *T. xiphioides*, *T. crocata* and *T. caliginosa*.

#	Family	Compound	RI	m/z	RT (min)	Mean ±SD (x10⁶)					Odor characteristics[f]
						YC	OC	Ca	LP-xiphi	HP-xiphi	
**1**	**M**	**α-thujene[b,d]**	**919**	**136**	**14.15**	nd	1.8 ±0.4	nd	3.2 ±1.8	nd	wood, green, herb
2	M	α-pinene[c,d]	927	136	14.46	2.5 ±0.6	2.9 ±0.4	2.4 ±1	4.5 ±1.2	nd	pine, turpentine
3	M	Sabinene[c,d]	969	136	16.05	2.1 ±0.4	3 ±0.3	2.7 ±0.9	3 ±1.5	nd	pepper, turpentine, wood
4	M	β-phellandrene[b,d]	973	136	16.23	2.5 ±0.4	5 ±0.5	4.2 ±2.1	4.6 ±1.1	nd	mint, terpentine
5	M	β-pinene[c,d]	979	136	16.46	nd	2 ±0.5	2.8 ±0.8	2.9 ±1.6	nd	pine, resin, turpentine
**6**	**M**	**β-myrcene[c,d]**	**988**	**136**	**16.78**	**4.6 ±0.9**	**11.4 ±1.8**	**12.2 ±3.9**	**45.9 ±19.2**	**40.1 ±12.6**	**balsamic, must, spice**
7	M	α-phellandrene[b,d]	1007	136	17.51	nd	nd	nd	6.5 ±5.2	nd	citrus, herbal, woody, peppery
**8**	**M**	**Limonene[c,d]**	**1033**	**136**	**18.52**	**58.6 ±8.5**	**107.5 ±12.4**	**81.1 ±24**	**99 ±44.8**	**69.7 ±71.5**	**lemon, orange**
**9**	**M**	**Eucalyptol[c,d]**	**1036**	**154**	**18.66**	**74.4 ±11.5**	**107.8 ±15**	**90.2 ±27**	**69.9 ±30.5**	**47.7 ±40.3**	**mint, sweet**
10	M	β-ocimene[c,d]	1045	136	18.98	nd	nd	3 ±1.2	183.8 ±212.7	56.3 ±104.8	sweet, herb
11	M	γ-terpinene[c,d]	1058	136	19.5	nd	nd	1.6 ±0.5	2.9 ±1.6	nd	gasoline, turpentine, lemon
12	M	Terpinolene[b,d]	1086	136	20.57	nd	nd	nd	3.3 ±1.7	nd	sweet, fresh, pine, citrus, woody, lemon peel
13	M	β-linalool[c,d]	1097	154	21.02	nd	nd	nd	15.8 ±10.9	97.3 ±38.7	citrus, orange, floral, terpy, rose
14	M	β-fenchol[c,d]	1171	154	23.8	nd	nd	2.3 ±0.5	nd	nd	pine, woody, dry, sweet, lemon
**15**	**M**	**α-terpineol[c,d]**	**1196**	**154**	**24.71**	**10.7 ±2.1**	**18.3 ±6**	**15.9 ±2.8**	**8 ±6.2**	**5.5 ±6.3**	**oil, anise, mint**
16	M	Geraniol[c,d]	1249	154	26.64	nd	2.9 ±0.6	nd	11.5 ±9.7	6.7 ±2.8	rose, geranium
17	M	Methyl geranate[b,e]	1320	182	29.02	nd	nd	nd	nd	11.4 ±7.5	waxy, green, fruity
**18**	**M**	**Geranyl acetate[c,e]**	**1378**	**196**	**30.59**	**3 ±1.3**	**2.5 ±0.3**	**1.5 ±0.4**	**191.3 ±103.6**	**56.5 ±40.1**	**floral, rose, lavender, green, waxy**
19	M	Geranyl butyrate[b,e]	1556	224	34.75	nd	nd	nd	14.7 ±10.5	7.7 ±7.4	fruity-floral, rose, apple-like
20	M	Geranyl tiglate[b,e]	1695	236	37.39	nd	nd	nd	nd	8.2 ±9.8	sweet-herbaceous, geranium-like, light fruity undertone
21	M	Geranyl hexanoate[b,e]	1748	252	38.27	nd	nd	nd	9.3 ±6.7	7.4 ±7.4	rose, geranium, fruity, waxy
22	S	α-bergamotene[b,e]	1439	204	32.16	1.8 ±0.5	nd	nd	5.3 ±3.2	5.1 ±4.6	wood, warm, tea

**23**	**S**	**β-farnesene[b,e]**	**1454**	**204**	**32.55**	**50.9 ±22.3**	**11 ±1.7**	**7.2 ±3.8**	**32.1 ±13**	**20.9 ±8**	**wood, citrus, sweet**
**24**	**S**	**α-farnesene[b,e]**	**1503**	**204**	**33.69**	**10.8 ±4.7**	**4.1 ±0.9**	**2.6 ±1.2**	**11.8 ±6.2**	**7 ±4.3**	**wood, sweet**
25	S	β-bisabolene[b,e]	1512	204	33.86	3 ±0.9	1.9 ±0.3	nd	8 ±2.8	10.1 ±4.6	balsamic, woody
26	S	α-bisabolene[b,e]	1544	204	34.52	nd	nd	nd	5.2 ±2.4	5.6 ±7.5	balsamic, spicy, floral
27	S	Nerolidol[c,e]	1567	222	34.98	2.5 ±0.8	4.2 ±4.6	nd	182.4 ±87.9	88 ±67.3	floral, green, waxy, citrus, woody
28	S	Denderalasin[b,e]	1577	218	35.17	nd	nd	nd	45.7 ±31	30.3 ±25	-
29	S	α-patchoulene[b,e]	1595	204	35.55	3.5 ±1.4	nd	nd	6.5 ±3.3	5 ±4.1	wood, spicy
30	S	Farnesol[c,e]	1720	222	37.81	85.1 ±27.2	40.1 ±6.6	13.7 ±6.7	nd	nd	fresh, green, muguet note
31	S	Farnesal[b,e]	1740	220	38.16	5.3 ±1.9	2.4 ±0.2	nd	nd	nd	floral, minty
32	S	Farnesyl acetate[b,e]	1832	264	39.67	41.2 ±18.7	4.9 ±1.5	9.9 ±8	nd	nd	green, floral, rose
33	P	Benzaldehyde[c,d]	962	106	15.8	4.6 ±3.2	2.2 ±0.1	1.6 ±0.2	nd	nd	fruity, powerful, cherry, bitter almond
34	P	Phenylacetaldehyde[c,d]	1043	120	18.92	21 ±49.3	2.4 ±0.5	nd	nd	nd	green floral, strongly hyacinth note
35	P	Methyl benzoate[c,d]	1094	136	20.89	7.5 ±3.2	2.9 ±0.5	16.4 ±8.7	nd	nd	aromatic, sweet, floral, a fruity undertone
36	P	Benzene ethanol[b,d]	1112	122	21.57	7.8 ±2.4	12.5 ±7.7	11.2 ±4.8	nd	nd	sweet, fresh, aromatic
37	P	Benzylacetate[c,d]	1161	150	23.43	50.2 ±23.3	nd	9.6 ±6.3	nd	nd	floral, fruity, jasmine, fresh
38	P	Methyl salicylate[c,d]	1193	152	24.63	5.2 ±2.5	10.4 ±2.9	17.7 ±7.3	nd	nd	minty, spicy, sweet
39	P	Phenylethyl acetate[c,d]	1255	164	26.87	130.4 ±52.3	nd	19 ±20.6	nd	nd	floral, rose, sweet, honey, fruity, tropical
40	P	Cinnamaldehyde[b,e]	1273	132	27.5	4.5 ±5	5.7 ±1.7	3.8 ±1.4	nd	nd	very strong, sweet, balsamic, typically cinnamon
41	P	Alcool cinnamique[c,e]	1305	134	28.61	10.9 ±16.7	10.8 ±2.9	6.3 ±3.6	nd	nd	warm-balsamic, floral-hyacinth, rose
42	P	Eugenol[c,e]	1353	164	29.96	8.2 ±7	136 ±49.4	91.8 ±20.7	nd	nd	sweet, spicy-clove, woody
43	P	Methyl eugenol[c,e]	1396	178	31.18	3.3 ±1.9	9.7 ±15.7	nd	nd	nd	spicy, cinnamon, clove, musty, vegetative, waxy, peppery
44	P	Cinnamyl acetate[b,e]	1446	176	32.37	nd	6.3 ±1.5	nd	nd	nd	sweet, floral, spicy, balsam, cinnamon
45	P	Isoeugenol[c,e]	1451	164	32.45	46.4 ±12.5	6.1 ±0.8	6.8 ±1.9	nd	nd	sweet, spicy, clove, woody
46	P	Methyl isoeugenol[b,e]	1494	178	33.49	26 ±23.8	8 ±13.1	nd	nd	nd	mildly spicy, like clove blossom oil
47	P	Zingerone[b,e]	1646	194	36.5	nd	4.6 ±1.9	20.1 ±9.3	nd	nd	sweet, spicy, phenolic, ginger, vanilla, woody
48	P	Benzyl benzoate[c,e]	1781	212	38.83	41.2 ±23.4	83.5 ±28.2	13.5 ±9.2	nd	nd	sweet, balsamic, floral, fruity nuances
49	P	Phenethyl benzoate[b,e]	1868	226	40.24	2 ±0.4	47.9 ±17.8	10.7 ±6.3	nd	nd	sweet, fruity, rose

50	P	Benzyl salicylate[b,e]	1885	228	40.51	15.5 ±9.9	47.9 ±23.6	nd	nd	nd	sweet, balsamic, slightly curative
51	P	Phenethyl salicylate[a,e]	-	242	42.09	nd	2.1 ±0.3	nd	nd	nd	balsamic, floral, rose, hyacinth carnation
52	O	Methyl hexanoate[b,d]	920	136	14.24	nd	nd	1.9 ±0.8	nd	nd	etheral, fruity, pineapple
53	O	Hexyl acetate[c,d]	1009	144	17.58	nd	nd	1.4 ±0.6	nd	nd	green, fruity, sweet, fatty, fresh, apple
54	O	Methyl nicotinate[c,d]	1139	137	22.59	121.9 ±24.4	143 ±28.9	80.9 ±11.8	nd	nd	warm, herbal, tobacco
55	O	Indole[b,e]	1296	117	28.22	10.6 ±6.6	5.6 ±3.1	nd	nd	6.6 ±6.7	mothball, burnt, animal, fecal
56	O	Methyl tetradecanoate[b,e]	1722	242	37.85	nd	nd	12.7 ±10.2	nd	nd	fatty, waxy, petal
57	O	p-tolyl octanoate[b,e]	1753	234	38.36	10.2 ±8.2	2.3 ±0.4	nd	nd	nd	animal, fecal, indole, green
58	O	11-hexadecenal[b,e]	1806	238	39.24	nd	nd	4 ±1.3	nd	nd	waxy
59	O	Hexadecanal[b,e]	1818	240	39.43	nd	nd	9.4 ±3.2	nd	nd	cardboard
60	O	Phenethyl octanoate[b,e]	1851	248	39.97	2.4 ±1	4.1 ±1.9	nd	nd	nd	sweet, waxy, green, cocoa, fruity
61	O	1-hexadecanol[b,e]	1879	242	40.42	18.1 ±8.6	9.5 ±4.7	76.3 ±9.2	nd	nd	waxy, greasy, floral oily
62	O	Methyl palmitoleate[a,e]	-	268	40.96	nd	nd	8.9 ±5.2	nd	nd	-
63	O	Methyl palmitate[a,e]	-	270	41.14	nd	40.6 ±20.2	12.8 ±10.7	nd	10.5 ±5.8	oily, waxy, fatty
64	O	Benzyl decanoate[a,e]	-	262	41.76	2.7 ±1.9	2 ±0.3	nd	nd	nd	-
65	O	11-hexadecenyl acetate[a,e]	-	282	42.43	nd	nd	5.4 ±1.3	nd	nd	-
66	O	Hexadecyl ethanoate[a,e]	-	284	42.66	4.9 ±2.8	nd	51.6 ±11.1	nd	nd	-

YC = Yellow *crocata*, OC = Orange *crocata*, Ca = *T. caliginosa*, nd = not detected., M = Monoterpene, S = Sesquiterpene, P = Phenylpropanoid, O = Other, a = identification performed by comparing the mass spectrum with that of the NIST library., b = identification performed by comparing the mass spectrum with that of the NIST library and by comparison of RI (retention index) with RI of published literatures and online library (https://webbook.nist.gov/chemistry/cas-ser.html), c = identification performed by comparing the mass spectrum with that of the NIST library, by comparison of RI (retention index) with RI of published literatures and online library and by comparison of retention time and mass spectrum of the authentic standard, d = efficient extraction with the first method, low values of temperature and extraction time (30 °C and 20 min), e = efficient extraction with the second method, high values of temperature and extraction time (75 °C and 65 min), f = odor characteristics were obtained from the "The Good Scents" company network database (www.thegoodscentscompany.com).
